# The effect of local anesthetics against planktonic forms and film formation of *S. aureus* strains and its dependence on antiseptics activity

**DOI:** 10.3389/fmicb.2023.1199899

**Published:** 2023-08-29

**Authors:** Mariia Faustova, Oleksandr Nazarchuk, Dmytro Dmytriiev, Yuliana Babina, Halyna Nazarchuk, Alina Dudar

**Affiliations:** ^1^Department of Microbiology, Virology and Immunology, Poltava State Medical University, Poltava, Ukraine; ^2^Department of Microbiology, National Pirogov Memorial Medical University, Vinnytsia, Ukraine; ^3^Department of Anaesthesiology and Intensive Care, National Pirogov Memorial Medical University, Vinnytsia, Ukraine; ^4^Department of Ophthalmology, National Pirogov Memorial Medical University, Vinnytsia, Ukraine

**Keywords:** surgical site infections, *Staphylococcus*, antiseptics, local anesthetics, biofilms

## Abstract

Today surgical site infections (SSIs) remain the second among hospital acquired infections in Europe and the USA. *Staphylococcus aureus* as a pathogen of nosocomial infections occur more frequently in surgical hospitals. The work was aimed to establish the effect of local anesthetics against planktonic forms and biofilm-formation of *S. aureus* clinical strains and the relationship between the sensitivity of *S. aureus* strains to local anesthetics and antiseptics *in vitro*. The antimicrobial activity of local anesthetics (0.5%, bupivacaine, 2.0% lidocaine, 0.375% ropivacaine) and antiseptics (decamethoxine 0.02%, chlorhexidine 0.05%) against clinical strains of *S. aureus* was observed and studied their ability to produce biofilms. The antimicrobial effect of local anesthetics was lower compared to antiseptics, but we observed inhibition of growth and reproduction of *S. aureus* in their presence. The ropivacaine solution and the lidocaine solution demonstrated almost the same activity against the studied microorganism isolates. Along with this, bupivacaine solution had the highest activity against the studied microorganisms. The minimal inhibitory concentration of bupivacaine for *S. aureus* was 2.2 times lower than the minimal inhibitory concentration of lidocaine and 2.1 times lower than the minimal inhibitory concentration of ropivacaine significantly (*p* < 0.05). Scientific research on various aspects of the formation of bacterial biofilms is a relevant area that will change approaches to the prophylaxis and treatment of a number of infections, including SSIs.

## 1. Introduction

The latest studies by the World Health Organization prove that surgical site infections (SSIs) are the most frequent nosocomial infections in low-and middle-income countries. Undoubtedly, the level of SSIs is lower in high-developed countries, however today it remains the second among hospital acquired infections in Europe and the USA (World Health Organization, 2018). There are 7 patients in developed countries and 15 in developing countries with infectious postoperative complications per every 100 hospitalized patients (European Centre for Disease Prevention and Control, 2017; Ananieva et al., 2018; World Health Organization, 2018).

According to the European Center for Disease Prevention and Control, Gram-positive cocci are the causative agents of SSIs in 47% of cases, and *Staphylococcus aureus* (*S. aureus*) takes the prominent first place among them (European Centre for Disease Prevention and Control, 2013; European Centre for Disease Prevention and Control, 2017). The frequency of postoperative complications in hospitals of various profiles caused by representatives of this type of microorganisms was 17% in 2013 with an increasing trend each year. Today, the incidence of staphylococcal infections among all nosocomial infections is about 40% of cases (European Centre for Disease Prevention and Control, 2013; Pal et al., 2019).

*Staphylococcus aureus* causes severe septic conditions in patients, a wide range of purulent superficial skin lesions and deep infections (Pal et al., 2019; Avetikov et al., 2020; Shaprynskyi et al., 2020). Such patients often require intensive therapy, the involvement of related specialists, and powerful courses of systemic antibiotics (Pérez et al., 2021). These prevalence and severity of the course of staphylococcal infections are associated with a powerful set of virulence factors of the pathogen and the rapid acquisition of antibiotic resistance (Cheung et al., 2021). Factors of adhesion and biofilm formation ensure the persistence of *S. aureus* on the tissues of the human body or implanted materials, creating additional foci of infection and providing the community of microorganisms with significant resistance to antiseptics and chemotherapeutic agents (Pałka et al., 2022). In turn, *S. aureus* is impressive in its speed of acquiring and spreading of multi-drug resistance. Over the past 10 years, the number of methicillin-resistant staphylococci has increased from 9 to 49%, infections which are characterized by complex long-term treatment and a high mortality rate (Pal et al., 2019; Cheung et al., 2021). On the basis of significant antibiotic resistance, the use of other substances, which potentially lead to antibacterial power and their successful combination in therapy of post-operative complications is promising.

Since the first mention of chlorhexidine in 1950, it has become the most widely used antiseptic in medicine, including dental surgery (Pałka et al., 2022). Most often, chlorhexidine is used in concentrations of 0.5 and 0.12% as a component of toothpastes, mouth rinses, postoperative gels, and other dosage forms widely available in pharmaceutical chains around the world. Due to this, its usage is beyond any control and frequently abused (Pitten and Kramer, 1999; Pałka et al., 2022). This situation led to a number of negative consequences, such as a decrease in the effectiveness of chlorhexidine and a general negative impact on the human health, especially during the COVID-19 pandemic, which increased sales of the above-mentioned antiseptic unreasonably (Johnson and Johnson Reports, 2021; Pałka et al., 2022). This dictates the need to review the principles of using of chlorhexidine and search for new effective antiseptic drugs.

Recently, antiseptics based on decamethoxine have become widely popular both in general and maxillofacial surgery. A number of scientific publications prove that the 0.02% solution of decamethoxine has a broad antimicrobial spectrum with no cytotoxic and no pro-apoptotic effects on the epithelium (Ivanova et al., 2019; Nazarchuk et al., 2019).

Local anesthetics have been used for years to provide surgical anesthesia or postoperative analgesia, the most common of which are lidocaine and bupivacaine. Recently, studies show their antimicrobial effect, although this mechanism of action has not yet been fully elucidated. Presumably, local anesthetics can inhibit the growth of living bacteria by reducing the membrane-dependent enzymatic activity of living cells, altering membrane permeability, and leading to ultrastructural changes. However, these findings have not been fully established and require additional research (Kesici et al., 2019).

Taking into account the fact that local anesthetics are constantly used in surgical stomatology in combination with antiseptics, and also have a limited antimicrobial effect, it is advisable to study their combined effect with the possible establishment of dependencies (Kesici et al., 2019).

The purpose of this study was to establish the effect of local anesthetics against planktonic forms and biofilm-formation of *S. aureus* clinical strains and the relationship between the sensitivity of *S. aureus* strains to local anesthetics and antiseptics *in vitro*.

## 2. Materials and methods

### 2.1. Specimen collection and culture isolation

Clinical isolates of *S. aureus* were obtained from patients with infectious postoperative complications, who were treated at the Municipal Enterprise “Poltava Regional Dental Center – Dental Clinical Polyclinics” of the Poltava Regional Council and Municipal Non-Profit Enterprise Vinnytsia Regional Clinical Hospital named after M. I. Pirogov Vinnytsia Regional Council during 2021–2022. Collection of samples was carried out with the sterile probe-tampons, which were placed in tubes with AMIES transport medium. Cultivation of microorganisms was performed on blood agar, salt agar, thioglycollate medium at a temperature of 37°C for 48 h. The final identification of isolates was carried out using an automatic bacteriological analyzer BIOMERIEUX VITEK 2 Compact (France), according to the manufacturer’s instructions.

In total, during the study, 28 clinical isolates of *S. aureus* were collected and identified from patients with infectious postoperative complications. All 28 isolates were tested for sensitivity to antiseptics and local anesthetics, and also for the ability to form biofilms in the presence of local anesthetics. The above studies were repeated three times.

Every patient had signed an informed consent for sampling of biological material and processing personal data before the start of the study. The study was performed in accordance with the Helsinki Declaration on Ethical Principles for Medical Research involving Humans (World Medical Association, 2013) and approved by the Bioethics Committee of National Pirogov Memorial Medical University, Vinnytsya, Ukraine (Protocol #10 10.11.22) and the Bioethics Commission of Poltava State Medical University, Poltava, Ukraine (#210, 23.11.22).

### 2.2. Antimicrobial agents

The sensitivity of *S. aureus* isolates was determined to local anesthetics: 2.0% lidocaine solution (PJSC Lekhim-Kharkiv, Kharkiv, Ukraine), 0.5% bupivacaine solution (DEMO SA Pharmaceutical Industry, Krioneri Attica, Greece) and 0.375% ropivacaine solution (Yuriia-Pharm LLC, Cherkasy, Ukraine). A 0.02% aqueous solution of decamethoxine (Yuriia-Pharm LLC, Kyiv, Ukraine) and a 0.05% solution of chlorhexidine bigluconate (PJSC Pharmaceutical Factory Viola, Zaporizhzhia, Ukraine) were used in the study. Antiseptics and local anesthetics were obtained from reliable commercial sources.

### 2.3. Determination of the effect of antiseptics and local anesthetics on planktonic microorganisms

The broth microdilution method in a 96-well microplate was used to determine the minimum inhibitory concentration (MIC) of antiseptics and local anesthetics. Two-fold serial dilutions of the studied drugs were prepared in Muller-Hinton broth according to the recommendations of ISO standard 20776-1:2019 (ISO, 2019). The inoculum was prepared by suspending of overnight culture of *S. aureus* in broth with a final concentration of microorganisms of 5×10^5^ CFUs/ml equivalent to a turbidity of 0.5 McFarland standard. As a positive growth control, one well was filled with 50 μL of antimicrobial agent-free medium. As a negative control – one well of the microplate was left un-inoculated. Microplate with prepared dilutions were incubated at a temperature of 35°C for 18 h, followed by determination of the optical density of the contents of the wells in comparison with the negative control using a spectrophotometer (wavelength 600 nm). The MIC was the highest dilution of the studied antimicrobial agent, that prevented visible growth of bacteria.

The study was conducted on 28 clinical isolates of *S. aureus* with each anesthetic and antiseptic separately and repeated three times to obtain a reliable result.

### 2.4. Determination of the effect of local anesthetics on the ability of microorganisms to form biofilms

The ability of clinical isolates of *S. aureus* to form biofilms and the effect of the studied antimicrobial agents on their immature biofilm were investigated using the microtiter plate method with sterile 96-well flat-bottomed polystyrene trays (Stepanović et al., 2007). The overnight culture of *S. aureus* was suspended in a sterile saline solution with a final concentration of microorganisms ~10^5^ CFUs/ml, what corresponds to a turbidity of 0.5 McFarland standard. The inoculum (20 μL) was introduced into the wells of tablets containing 170 μL of Tryptic Soy Broth with 1% glucose and 10 μL of a subbacteriostatic concentration of the studied, antimicrobial agents followed by incubation at 37°C for 24 h. Wells with 200 μL of nutrient medium were used as a negative control, and wells with 180 μL of nutrient medium and 20 μL suspension of the studied microorganisms were used as a positive control. The matured biofilms, after three-time washing with phosphate-buffered saline and fixing with Bouin solution, were stained with 150 μL of 2.0% crystal violet (Hucker formulation) for 15 min at room temperature. The ability to form biofilms was assessed using a spectrophotometer (570 nm) and expressed in Optical Density Units (ODU). The value of ODU <0.120 was evaluated as a low ability to form biofilms, 0.221–0.239 – as average, ODU >0.240 – as a high indicator.

The study was conducted on 28 clinical isolates of *S. aureus* with each anesthetic and repeated three times to obtain a reliable result.

The effect of anesthetics on the process of biofilm formation, instead of mature biofilm, was determined. Therefore, the tested solutions were introduced to the suspension of microorganisms at the beginning of the biofilm formation. Subbacteriostatic concentrations of local anesthetics, used in the study, were calculated as 1/4 of the MICs of anesthetics for the investigated microorganisms.

### 2.5. Statistical methods

Mean, standard deviation, median, minimum, maximum value of frequency and percentage were used for descriptive statistics. Student’s *t*-test was used to compare two normally distributed groups. Univariate variance analysis (ANOVA: single factor) was used to compare the results of three or more groups of data. The Bonferroni correction adjusted a significance level to control the overall probability of errors (false positive) for multiple hypothesis tests. The result was considered significant reliably if the *p*-value was less than 0.05.

The average level of biofilm formation of 28 isolates of the studied bacteria without the influence of any agents was taken as a control against which the results of the effect of local anesthetics on the biofilm formation of *S. aureus* were compared.

In order to determine the relationship between the sensitivity of *S. aureus* strains to antiseptics and local anesthetics, we determined the correlation coefficient (r-Pearson), the absolute value of which characterized the binding force. Statistical analysis was performed using statistical software IBM SPSS Statistics version 22.0.

## 3. Results

**Figure 1:**
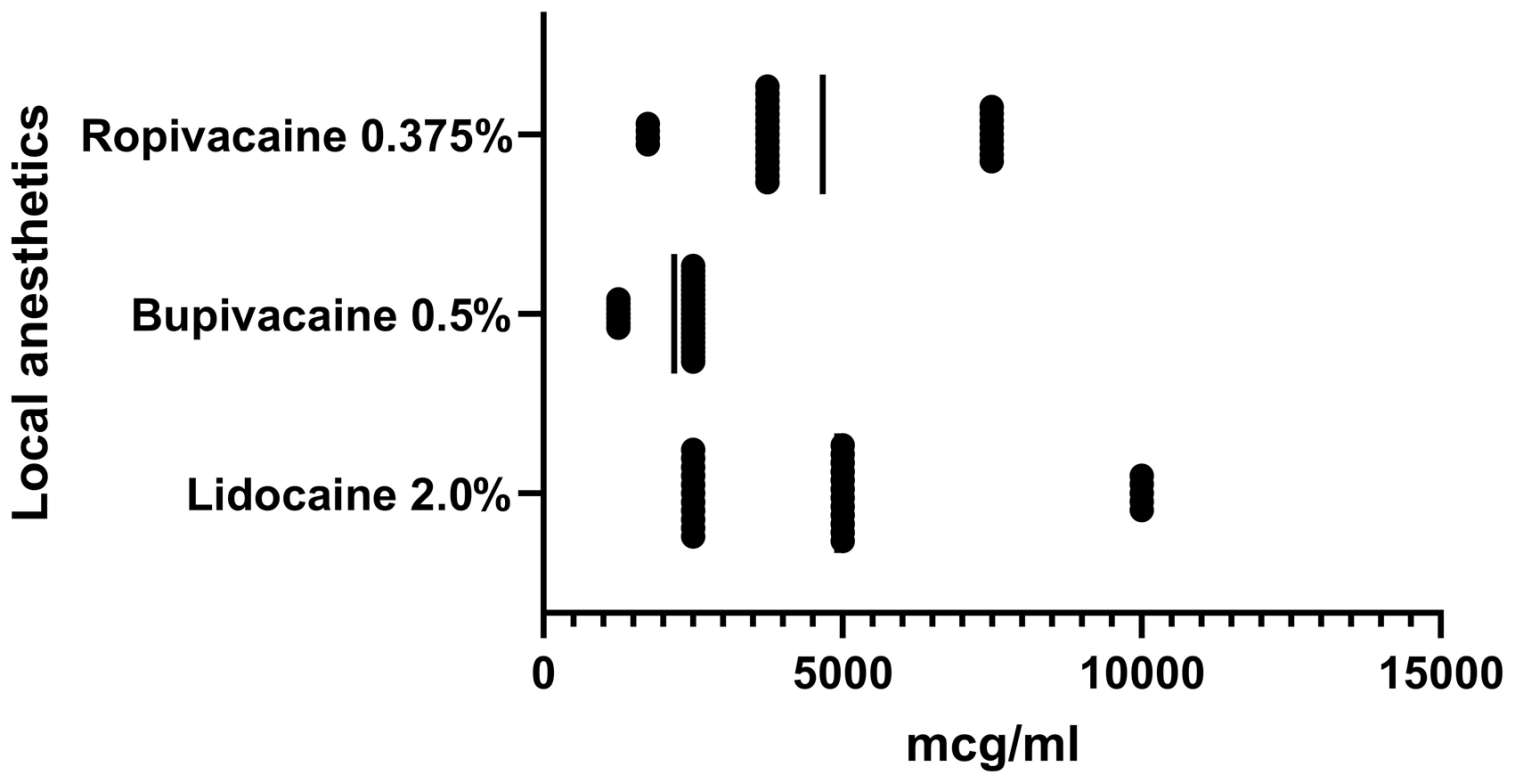
Graph shows the sensitivity of clinical isolates of *S. aureus* (*n* = 28) to local anesthetics, MIC, μg/ml, SD.

The sensitivity of clinical isolates of *S. aureus* isolated from patients with surgical complications to local anesthetics was established as the result of the research (Figure 1). We observed inhibition of growth and reproduction of planktonic forms of *S. aureus* in their presence. The ropivacaine solution and the lidocaine solution showed almost the same activity against the studied microorganism isolates. The average MIC of ropivacaine and MIC of lidocaine against *S. aureus* were 4.7 ± 2.1 mg/mL and 4.9 ± 2.7 mg/mL, respectively. Bupivacaine solution had the highest activity against the studied microorganisms. The MIC of bupivacaine (2.2 ± 0.6 mg/mL) for *S. aureus* was 2.2 times lower than the MIC of lidocaine and 2.1 times lower than the MIC of ropivacaine significantly (*p* < 0.05).

**Figure 2:**
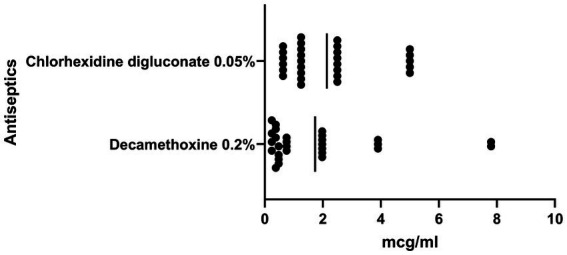
Graph shows the sensitivity of clinical isolates of *S. aureus* (*n* = 28) to antiseptics, MIC, μg/ml, SD.

The antimicrobial effect of antiseptics was higher compared to local anesthetics (Figure 2). The MIC of chlorhexidine bigluconate against to the *S. aureus* ranged from 5 μg/mL to 0.63 μg/mL and averaged 2.27 ± 1.59 μg/mL. In turn, the decamethoxine solution showed higher activity against *S. aureus* isolates compared to the chlorhexidine solution. The MIC of decamethoxine for the studied clinical isolates was significantly lower by 1.3 times (*p* < 0.05) and averaged 1.73 ± 2.06 μg/mL (ranged from 0.24 μg/mL to 7.80 μg/mL).

**Figure 3:**
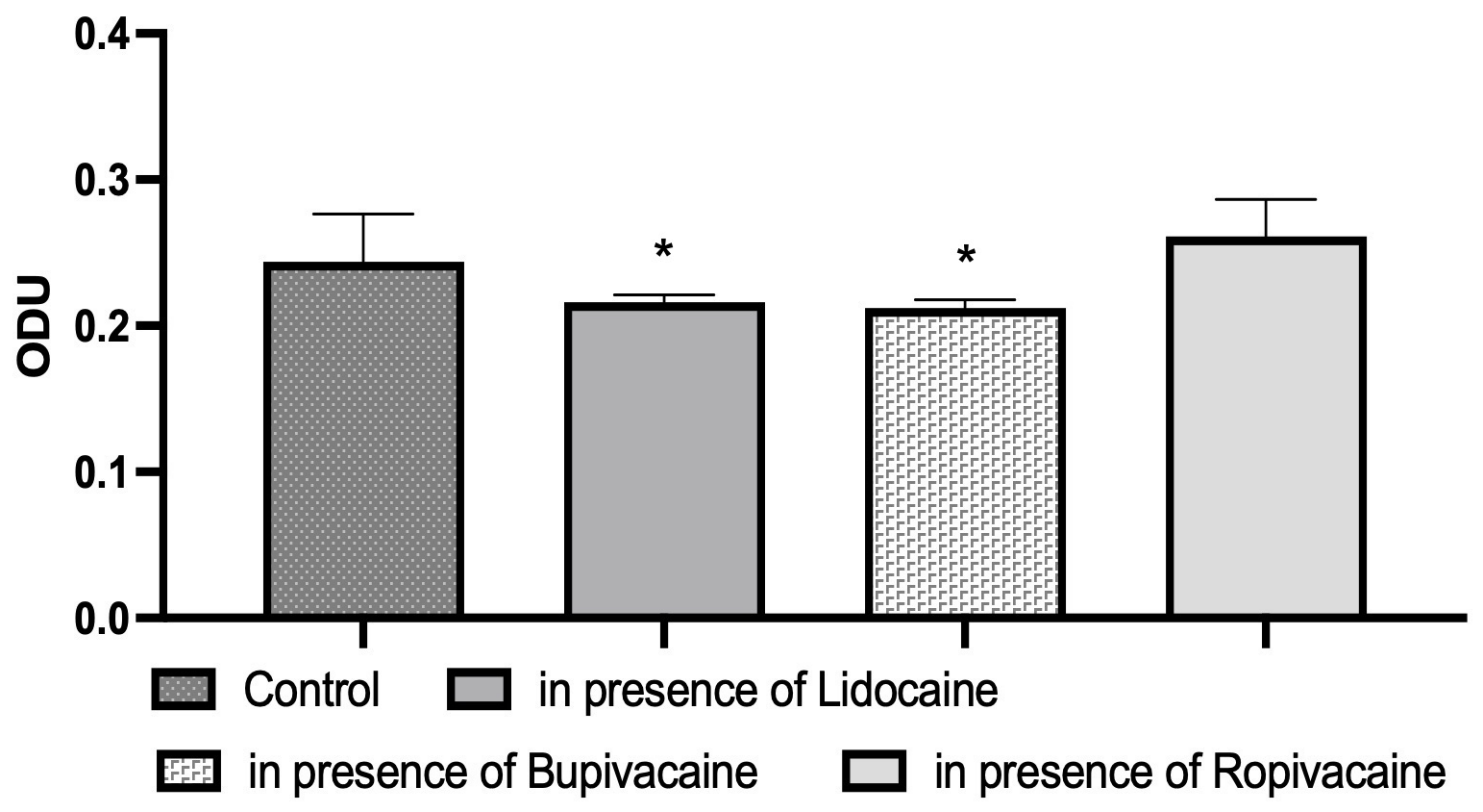
Graph shows the influence of local anesthetics on the ability of clinical isolates of *S. aureus* (*n* = 28) to form biofilms, ODU, SD [* – the reliability of the difference in the effect of a local anesthetic on the biofilm formation of *S. aureus* in relation to the control indicator (biofilm formation of *S. aureus* without any substances), *p* < 0.05].

It was established that local anesthetics had varying influence on the biofilm-forming properties of *S. aureus* (Figure 3). Subbacteriostatic concentrations of lidocaine and bupivacaine reduced the ability of the studied microorganisms to form biofilms significantly, compared to the anesthetic-free control. The initially high level of biofilm formation of *S. aureus* (0.244 ± 0.006 ODU), established during the study in control. This ability to form biofilms was lower in the presence of lidocaine by 1.1 times (12.0%), and in the presence of bupivacaine – by 1.2 times (13.0%) compared to the control (*p* < 0.05). During the cultivating of *S. aureus* biofilms with the above-mentioned anesthetics, its biofilm-forming ability was assessed as low, according to the criteria of interpretation of the used method. However, the subbacteriostatic concentration of ropivacaine did not reliably affect the biofilm-forming ability of clinical *S. aureus* isolates. The optical density of *S. aureus* biofilms formed in the presence of ropivacaine reached 0.261 ± 0.005 ODU.

**Figure 4:**
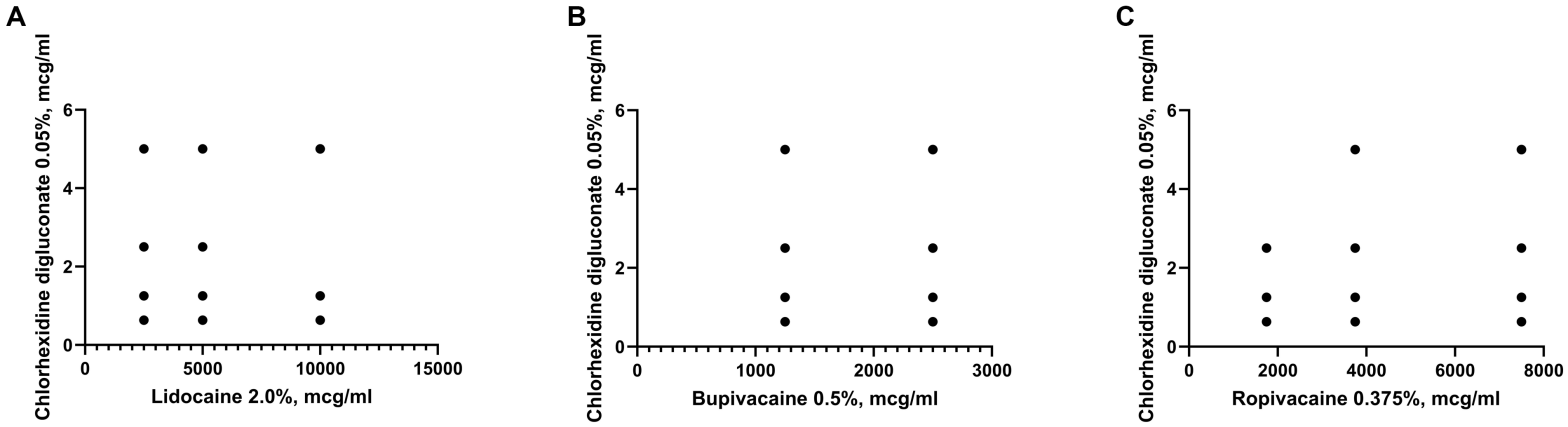
Graph shows the correlation between the sensitivity to Chlorhexidine bigluconate and local anesthetics: Lidocaine **(A)**, Bupivacaine **(B)**, Ropivacaine **(C)** of clinical isolates of *S. aureus* (*n* = 28).

It was established that the sensitivity of clinical isolates of *S. aureus* to local anesthetics did not depend on their sensitivity to Chlorhexidine bigluconate, since no reliable correlations were found between these parameters (Figure 4). The Pearson correlation coefficient between the sensitivity of the studied microorganisms to chlorhexidine and bupivacaine (*r* = 0.07) indicated a complete absence of correlation. In addition, correlation analysis of *S. aureus* susceptibility to chlorhexidine bigluconate and local anesthetics ropivacaine and lidocaine indicated a moderate correlation (*r* = 0.4), but the results were not statistically significant for both anesthetics.

**Figure 5:**
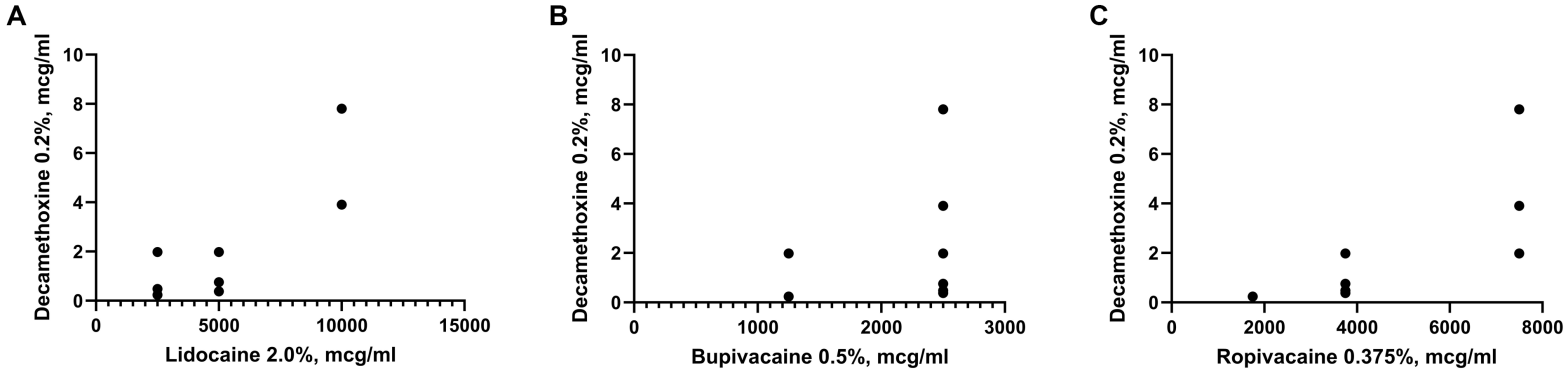
Graph shows the correlation between the sensitivity to Decamethoxine and local anesthetics: Lidocaine **(A)**, Bupivacaine **(B)**, Ropivacaine **(C)** of clinical isolates of *S. aureus* (*n* = 28).

The sensitivity of the studied microorganisms to bupivacaine also did not depend on the results of their sensitivity to decamethoxine (Figure 5). The Pearson correlation coefficient between the above indicators was *r* = 0.2. In turn, we found a reliable direct high correlation between the sensitivity of clinical isolates of *S. aureus* to decamethoxine and local anesthetics lidocaine and ropivacaine. This was indicated by high Pearson correlation coefficients between the sensitivity of the studied isolates to decamethoxine and local anesthetics: lidocaine *r* = 0.8 (*p* < 0.0001) and ropivacaine *r* = 0.7 (*p* < 0.0001).

## 4. Discussion

Taking into account the rapid spread of antibiotic resistance among microorganisms, which play the leading role in the development of postoperative complications, the search for new drugs or new combinations of already known agents are priority directions for combating this problem (Furusawa et al., 2018; World Health Organization, 2018). Undoubtedly, antiseptics and local anesthetics are used in combination with antibiotics in the treatment of infectious complications in order to prevent wound contamination and control pain (Kesici et al., 2019). However, if the antimicrobial effect of antiseptics is not in doubt, then the antimicrobial effect of local anesthetics needs further study. The first data on the possible antibacterial effect of local anesthetics appeared as early as the beginning of the 20th century, but even today there are limited studies on this field and they vary significantly, and sometimes even contradict each other. Therefore, a thorough study of the impact of local anesthetics on the microbiota of various human biotopes requires further study (Tulgar et al., 2018; Kesici et al., 2019).

Earlier works on determining the sensitivity of staphylococci to local anesthetics noted the lack of antistaphylococcal action of lidocaine. However, there are a number of studies in recent years that indicate inhibition of the growth and reproduction of *S. aureus* strains under the action of this anesthetic, what, in fact, coincides with the results of this study (Lu et al., 2014; Srisatjaluk et al., 2016; Kesici et al., 2019; Dmytriiev, 2020). A number of authors indicate the absence of an inhibitory effect of ropivacaine against Gram-positive and Gram-negative bacteria as well as yeasts (Aydin et al., 2001). In addition, some antibiotics are able to modestly reduce the clearance of ropivacaine by inhibiting CYP1A2-mediated formation of 3-OH-ropivacaine. It has been established that simultaneous use of ciprofloxacin with ropivacaine can cause toxicity (Jokinen et al., 2003). The results obtained by us also demonstrate the lowest antistaphylococcal effect of ropivacaine, what reduces its prospects for combined use in the treatment of postoperative complications. The number of publications on the sensitivity of staphylococci to bupivacaine is quite limited (Adler et al., 2017), although according to the results of our study, this local anesthetic showed the best antimicrobial properties. Despite the fact that the studied local anesthetics belong to the same aminoamide group, bupivacaine and ropivacaine, unlike lidocaine, contain terminal amino portions in the middle of the piperidine ring. That is why, thanks to the difference in the chemical structure, different pharmacological effects are possible, including antimicrobial activity (Viderman et al., 2021; Nazarchuk et al., 2022).

It is known that bacteria in the biofilm pose a much greater threat to human life and health (Kesici et al., 2019). Therefore, the key to successful treatment of infectious postoperative complications is the use of antimicrobial agents that affect not only planktonic forms of bacteria, but also their biofilmforming potential. Our results indicate a reduction in biofilm formation by *S. aureus* isolates in presence of bupivacaine and lidocaine, which should be taken into account during the planning of analgesia and treatment of patients. However, the sensitivity of *S. aureus* to bupivacaine does not depend on their sensitivity to the decamethoxine and chlorhexidine bigluconate, which makes impossible the predicted double antimicrobial effect on microorganisms from both the antiseptic and anesthetic side during their combination. It follows that among the studied local anesthetics, only lidocaine had an antistaphylococcal effect followed by strong dependence in increasing of the sensitivity to it among *S. aureus* isolates with the sensitivity to decamethoxine. That is, it creates prerequisites for their combined successful use in the management of patients with infectious postoperative complications caused by representatives of the *S. aureus* species. After all, determining the direct relationship between the sensitivities of pathogens to antiseptics and anesthetics at the same time is a rather promising direction in the fight against antibiotic-resistant isolates in the treatment of post-operative complications.

## 5. Conclusion

Among local anesthetics of the amide group, bupivacaine showed the highest antibacterial effect against clinical isolates of *S. aureus*, however, the level of sensitivity of microorganisms to it has no correlation with their sensitivity to decamethoxine and chlorhexidine bigluconate. The sensitivity of *S. aureus* to ropivacaine and lidocaine are highly correlated with their sensitivity to decamethoxin solution, what makes them promising in combined use. In addition, lidocaine and bupivacaine reliably inhibit the formation of *S. aureus* biofilms.

### 5.1. Limitations of the study

The study was conducted during the COVID-19 pandemic, which may have affected the results due to the increased use of antibiotics and antiseptics.

The study only included bacteria from the patients of two hospitals, which may limit the generalizability of the findings to other clinics.

The study only considered a limited number of local anesthetics and antiseptics and did not explore the effectiveness of others.

## Data availability statement

The raw data supporting the conclusions of this article will be made available by the authors, without undue reservation.

## Ethics statement

The studies involving humans were approved by Bioethics Committee of National Pirogov Memorial Medical University, Vinnytsya, Ukraine (Protocol #10 10.11.22) and the Bioethics Commission of Poltava State Medical University, Poltava, Ukraine (#210, 23.11.22). The studies were conducted in accordance with the local legislation and institutional requirements. The participants provided their written informed consent to participate in this study.

## Author contributions

DD and ON: conceptualization. MF and HN: methodology. MF and AD: software. ON: formal analysis, data curation, and supervision. MF and YB: investigation and writing—original draft preparation. AD: resources. MF: writing—review and editing. YB and HN: visualization. DD: project administration. All authors have read and agreed to the published version of the manuscript.

## Conflict of interest

The authors declare that the research was conducted in the absence of any commercial or financial relationships that could be construed as a potential conflict of interest.

## Publisher’s note

All claims expressed in this article are solely those of the authors and do not necessarily represent those of their affiliated organizations, or those of the publisher, the editors and the reviewers. Any product that may be evaluated in this article, or claim that may be made by its manufacturer, is not guaranteed or endorsed by the publisher.
